# Efficacy of lateral- versus medial-approach hip joint capsule denervation as surgical treatments of the hip joint pain; a neuronal tract tracing study in the sheep

**DOI:** 10.1371/journal.pone.0190052

**Published:** 2018-01-12

**Authors:** Waldemar Sienkiewicz, Agnieszka Dudek, Krzysztof Czaja, Maciej Janeczek, Aleksander Chrószcz, Jerzy Kaleczyc

**Affiliations:** 1 Department of Animal Anatomy, Faculty of Veterinary Medicine, University of Warmia and Mazury, Olsztyn, Poland; 2 Veterinary Biosciences & Diagnostic Imaging, College of Veterinary Medicine, University of Georgia, Athens, GA, United States of America; 3 Department of Animal Anatomy, Faculty of Veterinary Medicine, Wroclaw University of Environmental and Life Sciences, Wrocław, Poland; Public Library of Science, UNITED KINGDOM

## Abstract

**Objective:**

To evaluate efficacy of denervation of the of the hip joint capsule (HJC), as a treatment of hip joint pain. Specifically, we tested the hypothesis that HJC denervation will significantly reduce the number of sensory neurons innervating the capsule.

**Study design:**

Denervation of the HJC from a medial or lateral approach was followed by retrograde tracing of sensory neurons innervating the capsule.

**Animals:**

Twenty adult male sheep (30–40 kg of body weight; Polish merino breed) were used in the study.

**Methods:**

The hip joint was denervated from medial (n = 5) or lateral (n = 5) surgical approaches. Immediately after denervation, the retrograde neural tract tracer Fast Blue (FB) was injected into the HJC. An additional ten animals (n = 5 for medial and n = 5 for lateral approach) received the same treatment without HJC denervation to provide the appropriate controls.

**Results:**

Results of the study revealed that the vast majority of retrogradely labelled sensory neurons innervating the HJC originate from fifth lumbar to second sacral dorsal root ganglia. Both the medial and the lateral denervations significantly reduced the number of sensory neurons innervating the HJC (39.2% and 69.0% reduction respectively).

**Conclusions:**

These results show that denervation of the HJC is an effective surgical procedure for reduction of the sensory neuronal input to the HJC. Moreover, the lateral approach was found to be significantly more effective for reducing sensory innervation as compared to the medial one.

## Introduction

Hip joint arthritis (HJA) is one of the most common orthopaedic problems in both humans and dogs. It is accompanied by severe pain originating mainly from the richly innervated joint capsule [1–4]. A treatment based on denervation of the joint capsule may be a cost-effective alternative method for reduction of pain. For the patient, this means a significant improvement in the quality of life and more importantly, significant slowing of the atrophy of pelvic limb muscles [5–7]. Hip joint pain reduction can also be achieved using shockwave therapy [8;9], caloric restriction [10;11], physical activity control [12], physiotherapy [13;14] non-steroidal anti-inflammatory and analgesic drugs [15], or surgically. But long-term drug therapy carries a risk of side effects in the gastrointestinal tract, liver and kidneys [16].

The most common surgical procedures for hip dysplasia, including juvenile symphysiodesis (JPS), tripoint pelvic osteotomy (TPO), femoral head and neck ostectomy (FHO) and total hip replacement, have several limitations [17–19]. A foremost problem is the choice of procedure in older or obese animals that are not eligible for JPS or TPO. Hip prosthesis, in spite of its obvious advantages, in turn, has the disadvantage of being extremely expensive, but it is also fraught with high risk of complications reaching 20% of cases [20]. Therefore, denervation of the HJC, proposed in 1997 by Kinzel and Küpper for therapy of hip joint dysplasia or arthrosis in dogs, is a cost-effective surgical procedure that allows pain relief and carries very few limitations. It is based on transection of nerve branches supplying the hip joint capsule, which leads to analgesia, allowing painless and correct encumbering of the joint and thus prevents muscle atrophy [21]. Previous reports indicate a high efficacy of this procedure, reaching over 90% for more references see: [7, 22]. But these data are based mainly on subjective assessment of the owners of the animals. Studies using ground reaction force technique showed improvement in smaller percentages of patients [22–24]. One report showed the total ineffectiveness of minimally invasive denervation of the hip joint of in dogs [25] but unlike previous reports on hip dysplasia in this study pain and lameness were evoked by diffuse synovitis induced with injection of sodium urate into the right hip joint. Unfortunately, these collective studies were performed on small groups of animals (up to 10) making it difficult to draw final conclusions on significant effects. Toward that end, the authors of these reports themselves suggested the need to increase the number of individuals in studied groups or to prolong patient follow-up.

Currently, two surgical approaches of denervation of the HJC are commonly used in dogs. The first is a lateral approach and involves disrupting nerves supplying the HJC by removal the periosteum at the dorsolateral surface of the body of ilium and pelvic acetabulum [21;23;26;27]. The second is a medial approach and disrupts the nerves supplying the HJC by removal the periosteum at the ventral surface of the pelvic bone, ventral to the pelvic acetabulum [28]. The lateral approach denervates articular branches of the cranial gluteal and sciatic nerves, supplying the anterolateral region of the joint capsule [14]. The medial approach denervates articular branches of the femoral and obturator nerves [28]. Although the efficacy of the medial treatment is quite high a percentage of animals undergoing such denervation of the HJC fail to restore proper function of the joint. This has been suggested to result from insufficient abolition of pain sensation, or diverse hip joint pain cause (e.g., pain originating from the induced diffuse synovitis evoked by use of sodium urate crystal that influence on the efficacy of the denervation procedures [25]). The severity of degenerative joint diseases can also affect the efficacy of therapy [29].

The choice of method of surgery by veterinary practitioners is often dictated by subjective evaluation of the effectiveness of previously performed treatments, or by the selection of less complicated methods of surgery. It is very difficult to carry out the assessment of the effectiveness of the cranio-dorsal or ventral capsular neurectomy performed respectively from the lateral or medial approach which is based chiefly on subjective observations and information provided by the animal owners. Several published reports provide an objective assessment of the effectiveness of HJC denervation [22–25;30]. Among these reports, a parametric assessments, showed that 50% of the operated dogs demonstrated no post-operative progress [22–24] in spite of nearly all owners reporting health progress in their animals. Understanding this discrepancy in the evaluation of outcomes of a HJC denervation is critical for selecting the most effective treatment of hip joint arthritis. Therefore, the present study evaluated efficacy of medial and lateral approach denervation of HJC in sheep by a retrograde neural tract tracing method. The sheep animal model was used in the study because of its adequate size and anatomy of the hip joint, similar to both the dog and human [31]. We tested the hypothesis that HJC denervation will significantly reduce the number of sensory neurons innervating the capsule.

## Material and methods

Twenty male sheep of Polish merino breed were purchased from a breeding farm (6 months old, 30–40 kg of body weight) and randomly divided into four groups (n = 5). Five animals per group were used in the study, which was the calculated minimum necessary to test reliability for statistical significance in morphological studies. A power analysis was used to compute the animal number for analysis of variance. Based on our preliminary data and previous studies on phenotypes in peripheral ganglia, the number of animals was calculated to achieve a power of 0.80. All animal procedures were approved by the University of Warmia and Mazury (UWM) Institutional Animal Care and Use Committee (permission no. 86/2010) and conform to the National Ethics Commission for Animal Experimentation at the Polish Ministry of Science and Higher Education. Animals were housed in UWM-supervise animal facility in small ruminant stable with free access to food and water. Animal welfare was assessed by a facility veterinarian.

Experimental groups were: LC—lateral control, n = 5, LD–lateral denervated, n = 5, MC–medial control, n = 5, MD–medial denervated, n = 5. The hip joint denervation surgery was performed under general anaesthesia. The anaesthesia was achieved by an intravenous injection of ketamine (10 mg/kg BW; Bioketan^®^, Biowet Gorzów, Poland), diazepam (Relaninal^®^, Terpol, Poland, 0.02 mg/kg BW) and fentanyl (Fentanyl WZF, Polfa Warszawa, Poland 0.02 mg/kg BW,) mixture. During the surgery, 250 ml of 0.9% Natrium Chloratum in infusion was administered and the saturation and pulse were controlled using pulsoxymeter (Nonin^®^ Nonin Medical B.V., The Nederlands). In the LC and LD groups, the cranio-dorsal aspect of the hip joint was exposed according to modified Kinzel’s method [7;21].

**Fig 1:**
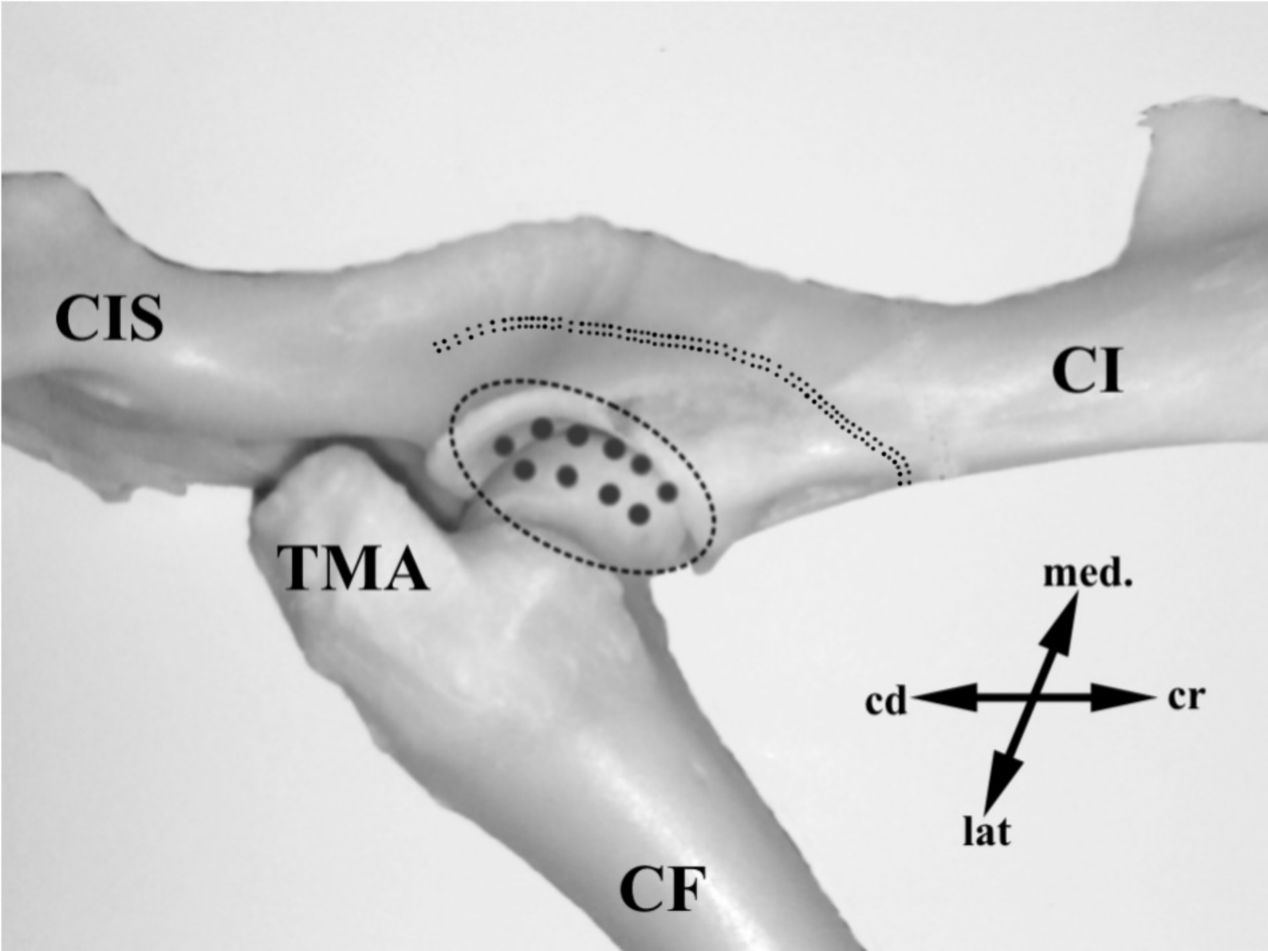
FB injection and denervation procedure–lateral aspect. Osteological specimen of the ovine hip joint–dorsolateral view. In the photograph the place of Fast Blue (FB) injection and the area of periosteum removal are drawn schematically. In animals of the lateral control (LC) and lateral denervated (LD) groups FB was injected into the lateral side of the HJC. The area of the HJC in which the tracer was injected is outlined with ellipse. The places of tracer injection are shown as grey spots in the delineated area. The area in which the periosteum was scraped with curette is indicated as a double dotted line. TMA, trochanter major; CF, corpus ossis femoris; CI, corpus ossis ilii; CIS, corpus ossis ischii; cr, cranial; cd, caudal; lat, lateral; med, medial.

To gain access to the HJC, a 5cm long skin incision was performed starting at the level of the greater trochanter and progressing toward the iliac crest, and then extended along the cranial border of the corpus of the femur to 1/3 of the length of the femur. Next, an incision through the *fascia lata* along the cranial border of *gluteobiceps* muscle was made. After dorsal retraction of the superficial, medial and deep gluteus muscles, the hip joint became visible. The neurectomy was performed by removal of the strip of periosteum from the lateral and craniodorsal aspects of the ilium above the acetabulum using a curette (Fig 1). Next, 20 μl of 5% sterile water suspension containing the retrograde tracer Fast Blue (FB; EMS-Chemie GmbH, Gross Umstadt, Germany) were injected using a 10 μl Hamilton syringe 801 RN (Hamilton CO., USA) into the cranio-dorsal aspect of the right HJC (10 microinjections, 2 μl each; Fig 1) to trace the sensory innervation. FB is a fluorescent dye most commonly used as a retrograde neuronal tracer, and is the tracer of choice for labeling in long-term experiments as it is effectively transported from periphery (target organ) to the perikaryon (the soma of neuron innervating the target organ) over long distances in various animal models [32]

To prevent the possibility of the leakage of the tracer the needle was placed into the joint capsule under a sharp angle, and tracer was injected over ten seconds to minimize backpressure. After withdrawal of the needle, the injection area was also cleaned with a sterile dry cotton swabs to remove any tracer excess.

**Fig 2:**
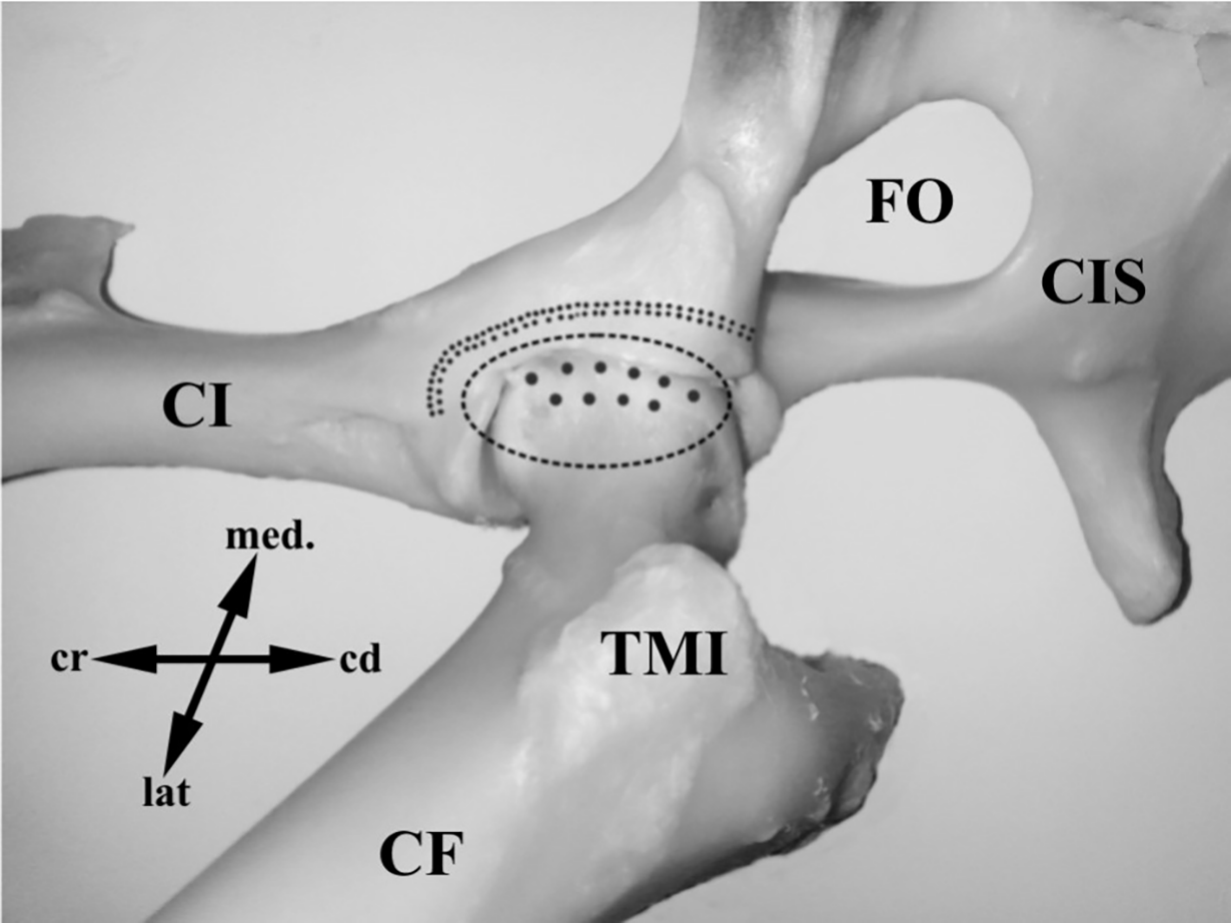
FB injection and denervation procedure–medial aspect. Osteological specimen of the ovine hip joint–ventromedial view. The place of FB injection and area of periosteum removal are shown schematically. In animals of the medial control (MC) and medial denervated (MD) groups, FB was injected into the ventromedial side of the HJC. The area of the HJC in which the tracer was injected is outlined with the ellipse. The places of tracer injection are shown as grey spots in the delineated area. The area in which the periosteum was scraped with a curette is indicated as a double dotted line. TMI, trochanter minor; CF, corpus ossis femoris; CI, corpus ossis ilii; CIS, corpus ossis ischii; FO, foramen obturatum; cr, cranial; cd, caudal; lat, lateral; med, medial.

In the MC and MD groups the neurectomy was performed form the ventral approach, according to modified Ballinari’s method [2]. using identical anaesthesia to the LC and MD groups. Briefly, the skin incision was done along the palpable pectineus muscle. After skin incision, the pectineus muscle was subtotally resected while performing optimal haemostasis. During the approach, the obturatorius nerve area was retracted caudally and the femoral vessels retracted cranially. The deep femoral vessels were preserved during the preparation of the origin of the pectineus muscle. The proximal caudal femoral vessels were retracted in a distal direction during transection of pectineus muscle, in the area of its musculotendinous junction. The deep femoral vessels were retracted and the hip joint capsule was exposed. The strip of the periosteum cranio-ventrally to the hip joint was removed using a curette (Fig 2). Next, intracapsular injection of the 20μl of 5% aqueous suspension (distilled water) containing sterile retrograde tracer FB was performed into the ventral aspect of the right HJC (groups MC and MD) in 10 injections of 2 μl each (Fig 2). The *fascia lata* (in the case of LC and LD groups) and subcutaneous tissue was closed using resorbable sutures (Vicryl 2.0, Ethicon, Poland), while the skin was closed using polyamide sutures (Amifil 2.0, Sinpo, Poland).

### Postoperative care

For three days after the surgery, the sheep were injected subcutaneously with meloxicam (Metacam^®^, Boehringer Ingelheim, Germany, 0.2 mg/kg of b.w.) to eliminate inflammatory symptoms such as pain and fever. The sheep also received the antibiotic enrofloxacin (Baytril^®^,Bayer, Germany, 5 mg/kg of b.w.) once a day for five days after the surgery to provide antimicrobial prophylaxis. Ten days after the surgery skin sutures were removed.

### Harvesting the tissues

After a 5-week survival period animals were euthanized by intravenous injection of sodium pentobarbital (Chemia, Gliwice, Poland; 100 mg/kg). Next the animals were transcardially perfused (chest was cut open reflecting the sternum cranially; the pericardium was cut open and the perfusion needle was inserted into the left ventricle of the heart and the right auricle of the heart was cut off) with 0.3 l of preperfusion solution containing 0.9% sodium chloride (Chemia, Gliwice, Poland), 2.5% polyvinylpyrolidone (Sigma, Deisenhofen, Germany), 0.5% procaine hydrochloride (Polfa, Warsaw, Poland), and 20 000 IU of heparin (Heparinum, Polfa; Poland, added extempore), followed by 4 l of 4% ice-cold buffered paraformaldehyde (pH 7.4). No traces of contamination with FB were found in the tissues, muscle tendons and skin surrounding the hip capsule. The epaxial muscles were excised to expose the vertebral column. The spinal cord and corresponding dorsal root ganglia were exposed via lumbo-sacral laminectomy. Next, the lumbar and sacral segments of the spinal cord (SC) including ipsi- and contra-lateral dorsal root ganglia (DRG), and the right hip joints were collected. The tissue samples were postfixed by immersion in 4% buffered paraformaldehyde (pH 7.4) for 30 min., rinsed with phosphate buffer (pH 7.4), and stored in 30% buffered sucrose solution (pH 7.4) until further processing. The SC-s and DRG-s were cut into 16-μm-thick cryostat sections. The sections were examined under a confocal microscope (LSM 700, Zeiss, Germany) equipped with a filter for FB. FB-labelled neurons in each DRG were counted in every third section (approximately 100 sections per ganglion) to eliminate the likelihood of counting the same neuron twice (Table A, B, C in S1 File). The SC-s were first cut into individual neuromers, sectioned and examined as described above. Because motoneurons of SC are much larger than sensory neurons in DRG-s, every sixth section from SC-s was investigated in search of FB+ neuronal cells. The resulting data were expressed as mean ± SEM and were analysed using a one-way ANOVA followed by a Holm-Sidak test for significance. Differences were considered to be statistically significant for P<0.05)

## Results

**Fig 3:**
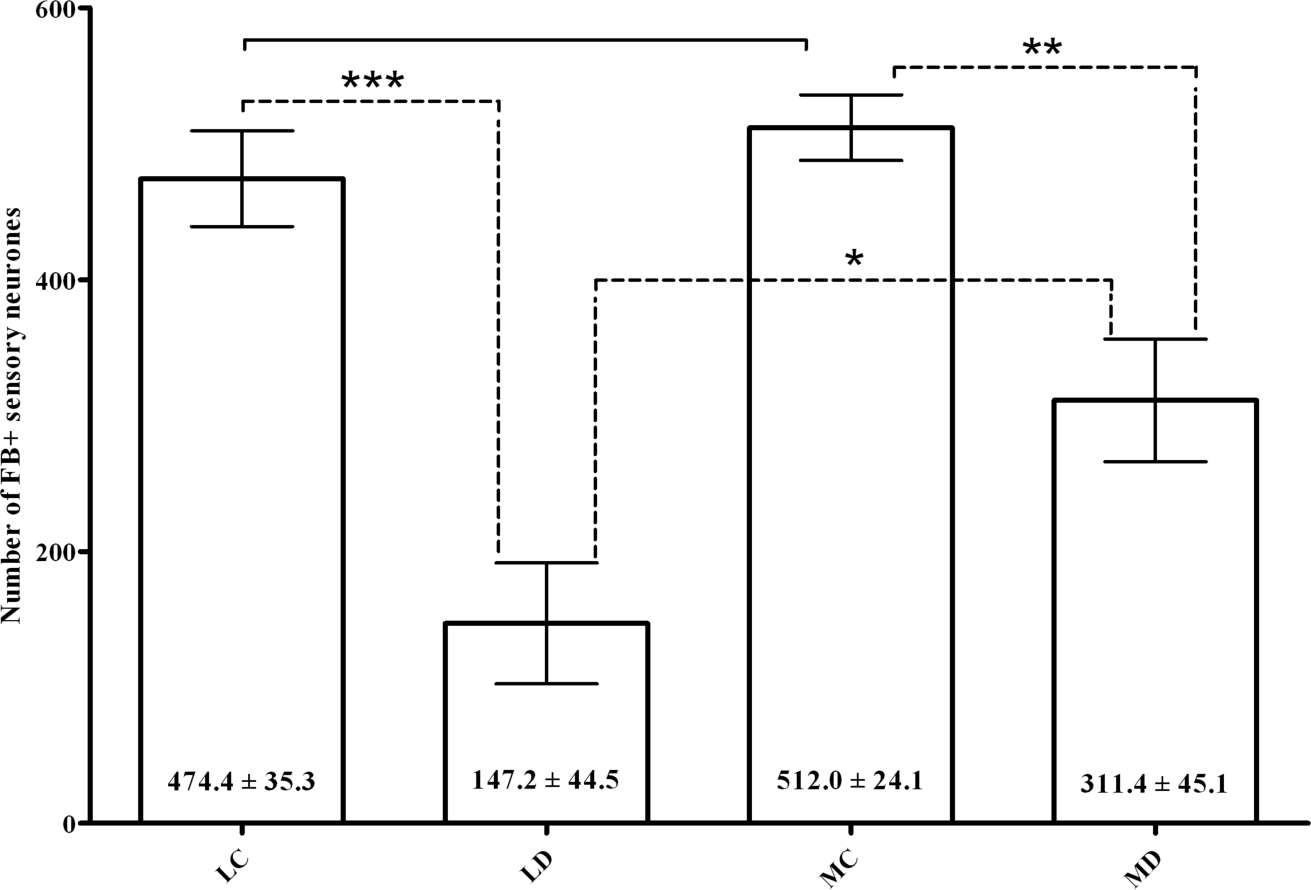
Graph showing the number of FB+ neurons and results of statistical analysis. The number of FB+ neurons per sheep in the control and experimental animals injected with FB into the lateral side of the hip joint capsule (HJC). Both the medial and the lateral approach of HJC denervation significantly reduced the number of remaining sensory neurons (FB+) innervating the capsule. The lateral approach was found to be significantly more effective for reducing sensory innervation as compared to the medial approach.

**Fig 4:**
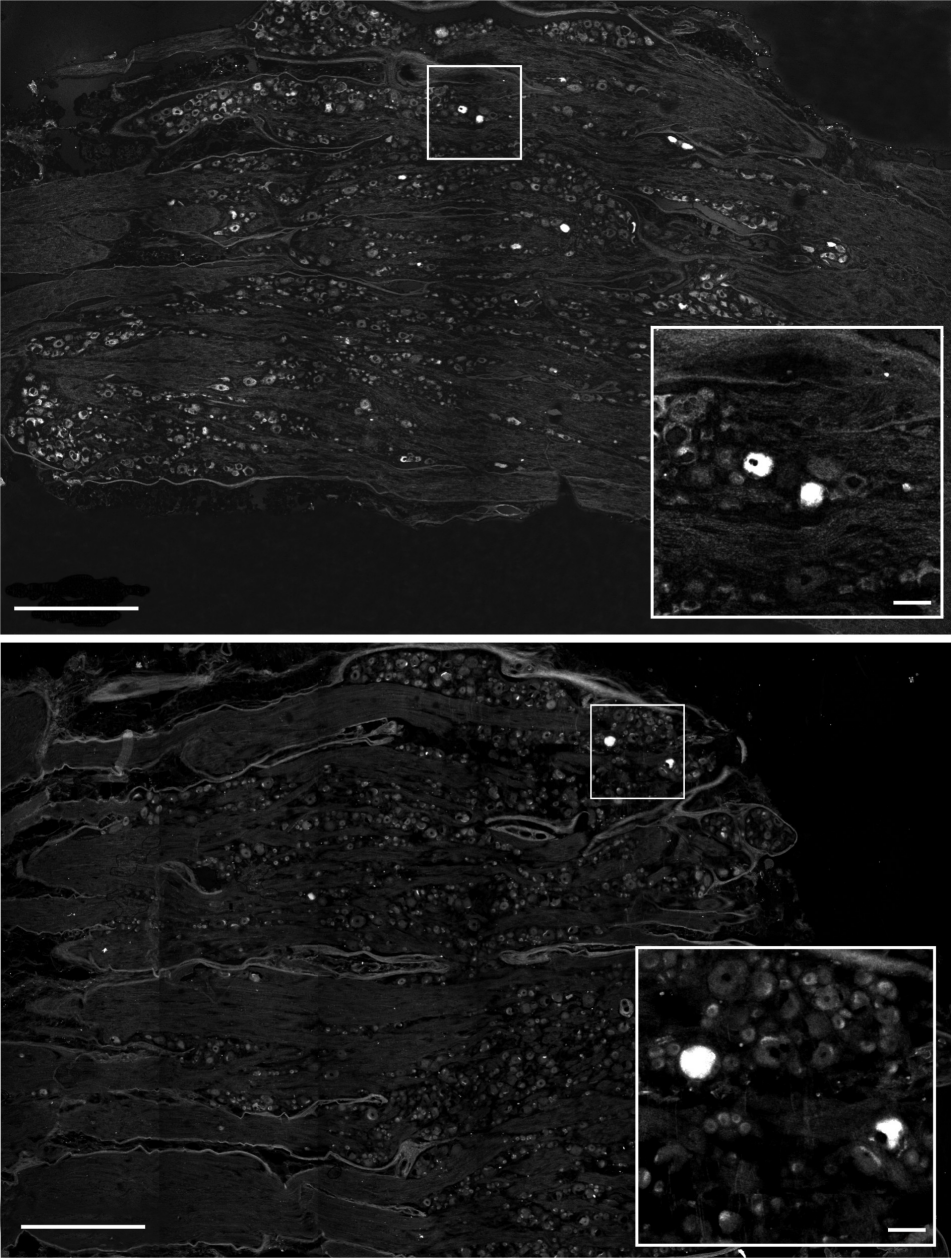
Microphotographs of the spinal ganglia. Longitudinal section of the sixth right lumbar spinal ganglion of the sheep of LC group injected with FB into the lateral part of the hip joint capsule (upper set). Lower set shows longitudinal section of the sixth right lumbar spinal ganglion of the sheep of LD group subjected to neurectomy and next injected with FB into the lateral surface of the hip joint capsule. Inserts show high magnification of retrogradely-labelled (FB+) sensory neurons innervating the capsule after the procedure. Photographs were captured by confocal microscope (LSM 700, Zeiss) as tiled scan. Scale bars: long = 1mm, short 100μm.

FB-positive neurons were found exclusively in the ipsilateral ganglia. The neuronal counts revealed no statistically significant difference (P = 0.4049) between the number of FB+ neurons observed in the LC and MC (control) groups and statistically significant differences between the number of FB+ in the LD and LD groups (P = 0.0004) and in the MC and MD groups (P = 0.0044) (Fig 3). The numbers of FB+ neurons in the LC and MC groups were 474.4 ± 35.3 and 512.0 ± 24.1 per animal, respectively. However, the counts in denervated animals were significantly different (P = 0.0320) in the number of FB+ neurons in DRG. In the LD and MD groups the number of FB+ neurons counted was 147.2 ± 44.5 and 311.4 ± 45.1 per animal respectively (Fig 3). The labelled neurons were round and oval in shape. They were scattered within the ganglia and did not form clusters (Fig 4).

**Fig 5:**
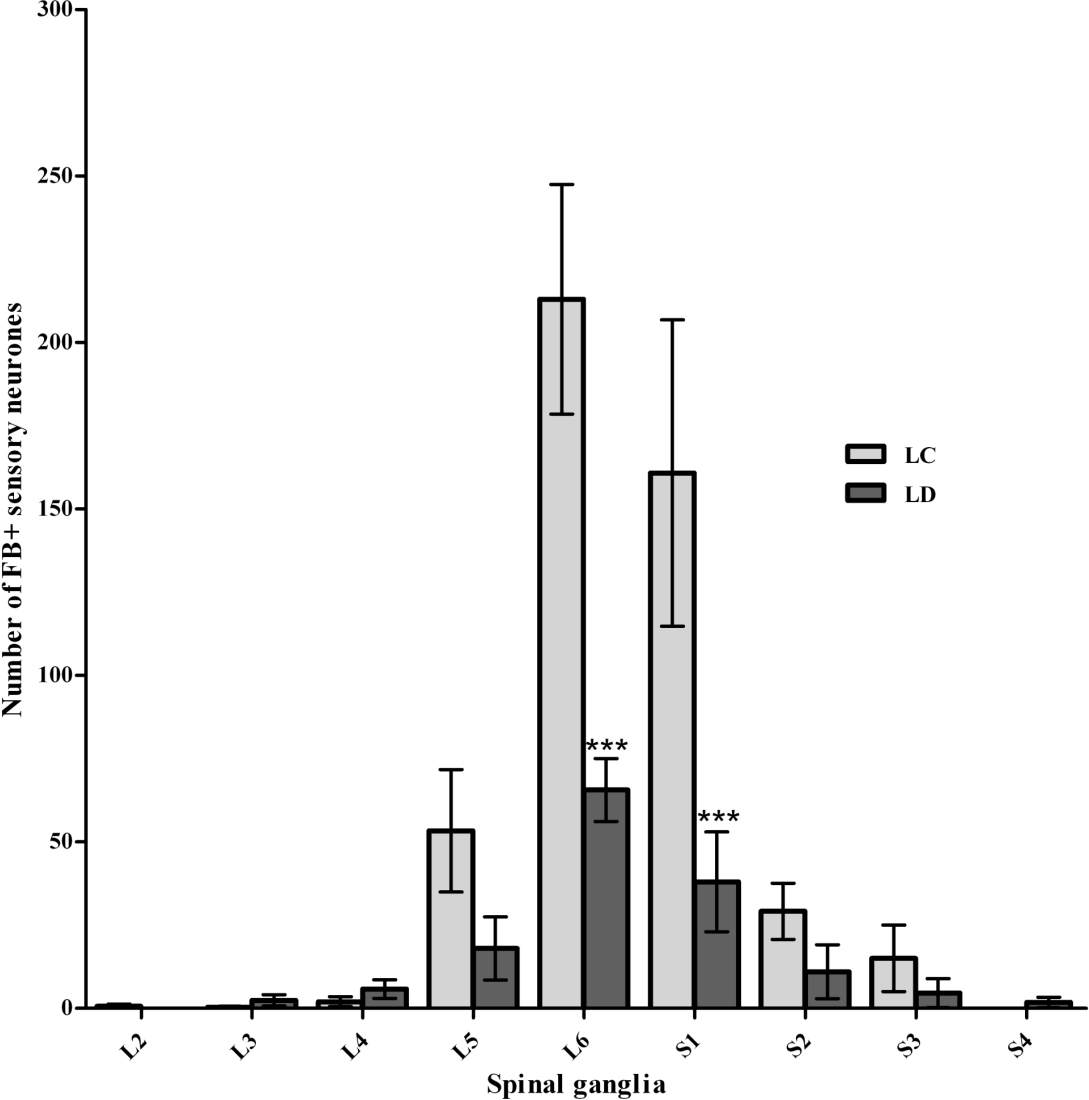
Graph showing the number of FB+ neurons and results of statistical analysis–lateral groups. The number of FB+ neurons in dorsal root ganglia in control and experimental sheep injected with FB in the lateral side of the HJC (after lateral denervation). HJC denervation from the lateral approach significantly decreased the capsule innervation originating from L6 and S1 dorsal root ganglia.

The analysis of cranio-caudal distribution of FB+ neurons in the DRG revealed, in the LC group, labelled neurons within the ipsilateral lumbar (L) and sacral (S) DRG from L2 to S3. Only solitary FB+ neurons were found in L2, L3, and L4 ganglia. The number of FB+ neurons was higher in L5 (53.3 ± 18.4, mean ± SEM) and the most numerous population of FB+ neurons was found in L6 (213.0 ± 34.5) and S1 (160.8 ± 46.0) (Fig 4). Smaller numbers of FB+ neurons were found in S2 and S3 (28.2 ± 8.4 and 15.0 ± 10.0 respectively). In LD animals, solitary FB+ neurons were observed in L3, L4 and L5 ganglia (2.4 ± 1.7, 5.8 ± 2.8 and 18.0 ± 9.5, respectively). The number of FB+ neurons in L6 and S1 was significantly smaller (P = 0.001) than that found in LC animals (65.6 ± 9.4 and 38.0 ± 15.0 respectively). In the S2, S3 and S4 ganglia solitary FB+ neurons were observed (11.0 ± 8.1, 4.6 ± 4.4 and 1. 8 ± 1.6 respectively; (Fig 5).

**Fig 6:**
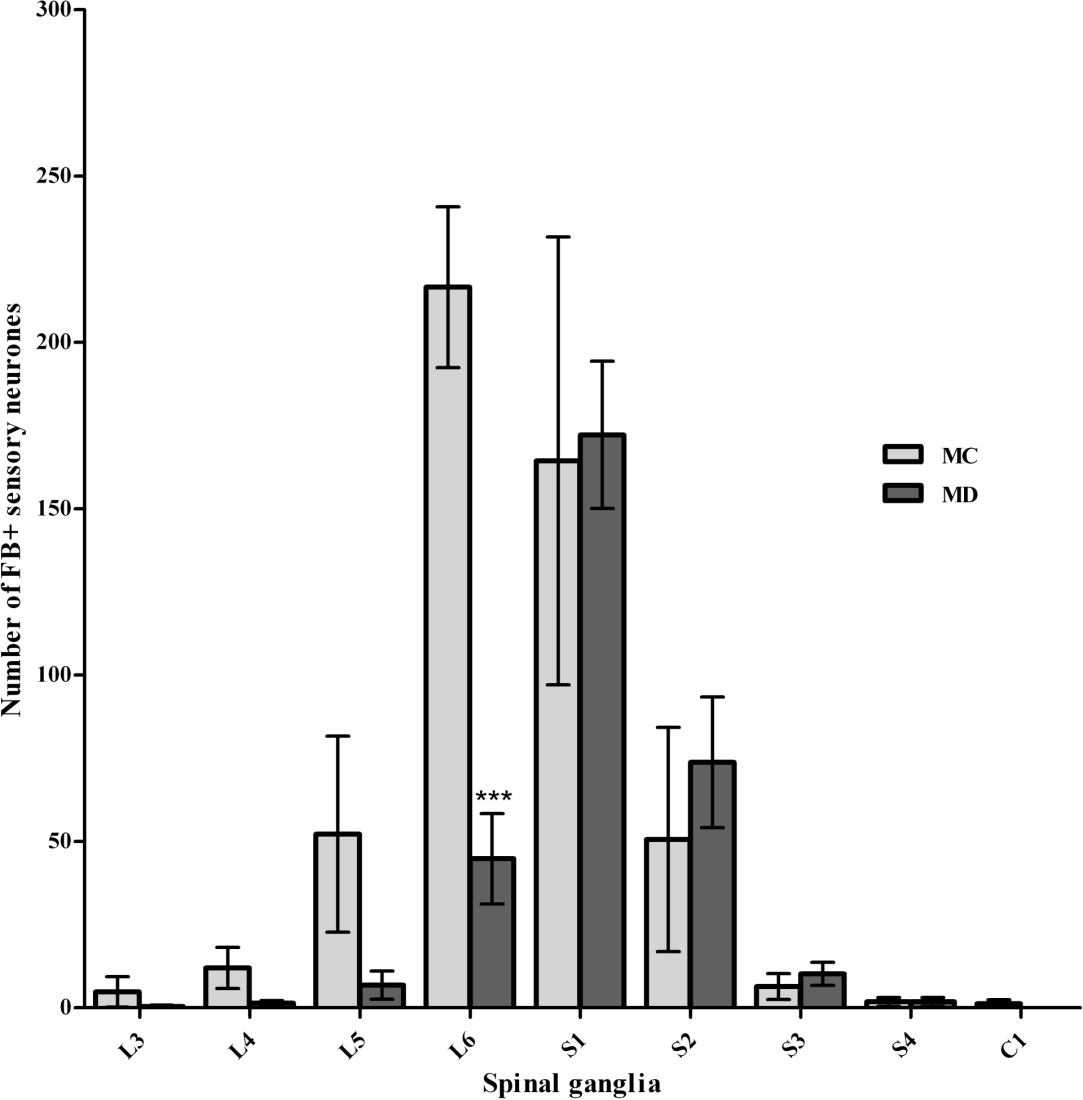
Graph showing the number of FB+ neurons and results of statistical analysis–medial groups. The number of FB+ neurons in dorsal root ganglia in control and experimental sheep injected with FB in to the medial side of the HJC (after medial denervation). HJC denervation from the medial approach significantly decreased the capsule innervation originating only from L6 dorsal root ganglion.

In the MC group, FB+ neurons were found in DRG at the lumbo-coccygeal level (L3 to C1; C = coccygeal). In the L3 and L4 ganglia only the solitary neurons were observed (4.8 ± 4.6 and 12.0 ± 6.2 of neurons respectively) (Fig 5). The L5, L6, S1 and S2 ganglia contained a much larger number of FB+ neurons (52.2 ± 29.5, 216.6 ± 24.7, 164.4 ± 67.3 and 50.6 ± 33.7, respectively). In the remaining (S3-C1) ganglia the average number of FB+ nerve cells was 6.4 ± 3.9, 1.8 ± 1.3 and 1.2 ± 1.2, respectively. In the MD group, a smaller number of FB+ neurons in L3-L5 ganglia was observed (0.4 ± 0.4, 1.4 ± 0.8, and 6.8 ± 4.2 respectively). The number of FB+ neurons in L6 ganglion was significantly smaller (P = 0.001) than that found in MC animals (44.8 ± 13.6). In the S1-S4 ganglia the number of FB+ neurons was 172.2 ± 22.1, 73.8 ± 19.7, 10.2 ± 3.5 and 1.8 ± 1.3, respectively (Fig 6). The studied sections of the spinal cord did not reveal FB-retrogradely labeled neurons.

## Discussion

Dissecting studies carried out on different species including humans [33–36] have disclosed four nerves contributing to the HJC nerve supply. The sciatic nerve gives off branches to the caudo-lateral part of the HJC in the dog [36–38] or the posterior and posterolateral part in humans [39]. The femoral nerve innervates the medial side of the canine HJC [36;37] or the anterior part in humans [39]. The postero-lateral part of the HJC in the dog and humans is also supplied by the cranial gluteal nerve [36–39]. The 4th nerve contributing to the innervation of the human HJC is the obturator nerve which supplies the antero-medial part of the human capsule [39].

In domestic and laboratory animal species, the participation of the obturator nerve in hip joint innervation has been described in horses [4] and in only two cats among many investigated [40]. No involvement of the obturator nerve articular branches in the innervation of the HJC was determined in the dog by Kinzel et al. [12]. However, Gasse et al. [9], using acetylcholine esterase staining, detected contributions of obturator nerve branches to the innervation of the caudal part of the HJC in the dog. The latter observation was confirmed by recent studies [36] describing short articular nerve fibres arising from the obturator nerve and supplying the caudal aspect of the HJC. Although former classical anatomical studies have disclosed nerves attaining the HJC directly, it has also been found that some sensory fibers can target the capsule indirectly, via its surrounding muscles and acetabular periosteum [1;41]. Our results expand the knowledge about the hip innervation and simultaneously confirm previous findings, particularly those reported recently [36].

The available literature offers only two contributions describing localization of sensory neurons supplying the hip joint. The first was performed in the rat and deals with a localisation of sensory neurons supplying the entire hip joint [42]. The second investigated the innervation of the lateral and medial part of the HJC in the rabbit [43]. Both studies were performed with the use of retrograde tracing. In the study performed in the rat, the labelled neurons innervating the entire hip joint were found within ipsilateral Th13 to L5 DRG. Studies in the rabbit by our laboratory have revealed neurons supplying the lateral side of the capsule in the ipsilateral L7 to S2 DRG, and those innervating the medial side of the HJC in the ipsilateral L6 to S4 DRG [43].

In the sheep, the traced neurons projecting to the lateral side of the HJC were determined within ipsilateral spinal ganglia from L2 to S3, whereas those supplying the medial side of the structure were distributed within ipsilateral ganglia from L3 to C1. The differences in the number and cranio-caudal distribution of the traced neurons [6 to 50 neurons per animal innervating entire hip joint in rat [42], 16 and 28 neurons per rabbit innervating lateral or medial side of HJC, respectively [43] and 474 or 512 neurons innervating the lateral or medial side of the HJC in the sheep] are probably at least largely due to different injection points, different volume of the tracer used and different counting methods. In the present study, the tracer was injected bilaterally (into two sides of the HJC, lateral and medial) and was administered in 10 microinjections, 2μl each, while in the rabbits [43] only 5μl of FB was injected in 5 microinjections, 1μl each. The lower amount of the tracer can result in the lower number of the traced neurons.

In the rat [42], only one injection of the tracer into the caudo-lateral side of the hip joint was performed. The tracer solution was spread throughout the entire hip including the joint capsule, synovial lining, bone and also the articular cartilage thus the results provided deal with sensory innervation of the whole joint, not only the joint capsule. The large number of traced neurons observed in the present investigation suggest the possibility of the leakage of the tracer resulting in the contamination of neighbouring tissues, however as mentioned in material and methods section measures were in place to prevent that.

As in a previous experiment [43] dimethyl sulfoxide (DMSO)was not added to the FB solution. Although DMSO added to the FB solution improves the uptake of the tracer by the nerve endings (which in present study was accomplished by a long survival time), it also augments FB penetration, which could result in the excessive infiltration and contamination of the adjacent tissues. The correctness of the tracer injections in our investigations was verified by the absence of the marked nerve cells in the spinal cord, which suggests that there was no leakage of the tracer into the neighbouring structures.

The distribution of neurons supplying the HJC in the spinal lumbar (L2-L6) and sacral (S1-S4) ganglia and even in the first coccygeal ganglion indicates the participation of the sciatic, femoral, cranial gluteal and obturator nerves in the innervation of the HJC. Denervation of the HJC from the lateral approach resulted in a distinct decrease in the number of the FB traced neurons in almost all the ganglia involved in the innervation of this structure. In this method of denervation, a strip of the periosteum on the lateral and dorsal aspect of the ilium was removed resulting in transection of the articular branches of sciatic and cranial gluteal nerves (innervating dorsal and cranio-lateral aspects of the HJC [36]. A decrease in the number of the traced neurons reached up to 70% (474 neurons per animal in LC group and only 147 neurons per animal in LD group). Denervation accomplished with the medial approach resulted in a decrease in the number of the traced neurons in the lumbar ganglia, whereas the number of the traced neurons in the sacral ganglia remained unchanged. The obtained results indicate that denervation of the medial aspect of the HJC results in damage of the articular branches of the femoral nerve supplying the cranio-ventral portion of the HJC [36], whereas branches of the obturator nerve supplying the caudal aspect of the HJC [36] remain intact.

## Conclusions

Our results show that denervation of the HJC is an effective surgical procedure for reduction of the sensory neuronal input to the hip joint capsule. Moreover, the lateral approach was shown to be more effective than the medial one. In conclusion, the results suggest that the lateral approach in denervation of the HJC should be preferred as an alternative treatment of hip joint pain in domestic animals. This conclusion is strongly supported by observations concerning the number of nerve fibres supplying the craniodorsolateral portion of HJC which is significantly greater than in caudolateral part of this organ [1] as well as a recently published report showing increased density of innervation of the HJC in the craniodorsolateral aspect of dogs with hip dysplasia [44].

## Supporting information

S1 Checklist ARRIVE checklist. (PDF)

S1 File Of neurones counted in individual animals and ganglia. **Table A.** The total number of FB-containing neurons in individual animals of control (LC and MC) and experimental (LD and MD) groups. **Table B.** Number of FB-containing neurons in particular ganglia in individual animals of control (LC) and experimental(LD) groups in which FB was administered to the lateral side of the HJC. **Table C.** Number of FB-containing neurons in particular ganglia in individual animals of control (MC) and experimental(MD) groups in which FB was administered to the medial side of the HJC.
(DOC)
