# Supplementary material for: Efficacy of lateral- versus medial-approach hip joint capsule denervation as surgical treatments of the hip joint pain; a neuronal tract tracing study in the sheep
Source: PLoS One. 2018 Jan 12;13(1):e0190052. doi: 10.1371/journal.pone.0190052 (PMC5766125; doi:10.1371/journal.pone.0190052)
Supplement: S1 File — Table A. The total number of FB-containing neurons in individual animals of control (LC and MC) and experimental (LD and MD) groups. Table B. Number of FB-containing neurons in particular ganglia in individual animals of control (LC) and experimental(LD) groups in which FB was administered to the lateral side of the HJC. Table C. Number of FB-containing neurons in particular ganglia in individual animals of control (MC) and experimental(MD) groups in which FB was administered to the medial side of the HJC. (DOC) [file pone.0190052.s002.doc]

Table A in S1 File.

| **Number of animal** | **LC** | **LD** | **MC** | **MD** |
| --- | --- | --- | --- | --- |
| **1.** | 445 | 101 | 596 | 334 |
| **2.** | 491 | 324 | 462 | 317 |
| **3.** | 584 | 94 | 534 | 237 |
| **4.** | 485 | 12 | 484 | 464 |
| **5.** | 367 | 95 | 484 | 205 |

Table B in S1 File.

| **LC** | | | | | | **LD** | | | | |
| --- | --- | --- | --- | --- | --- | --- | --- | --- | --- | --- |
| **Number of animal** | | | | | | **Number of animal** | | | | |
| **Ganglion** | **1.** | **2.** | **3.** | **4.** | **5.** | **1.** | **2.** | **3.** | **4.** | **5.** |
| **L2** | 0 | 0 | 3 | 0 | 0 | 0 | 0 | 0 | 0 | 0 |
| **L3** | 0 | 0 | 2 | 0 | 0 | 9 | 2 | 1 | 0 | 0 |
| **L4** | 0 | 3 | 7 | 0 | 0 | 11 | 14 | 3 | 0 | 1 |
| **L5** | 76 | 80 | 15 | 3 | 93 | 52 | 24 | 12 | 0 | 2 |
| **L6** | 211 | 300 | 272 | 107 | 175 | 95 | 54 | 74 | 66 | 39 |
| **S1** | 128 | 83 | 217 | 308 | 73 | 84 | 7 | 3 | 48 | 48 |
| **S2** | 28 | 25 | 14 | 60 | 14 | 43 | 0 | 1 | 6 | 5 |
| **S3** | 2 | 0 | 54 | 7 | 12 | 22 | 0 | 0 | 1 | 0 |
| **S4** | 0 | 0 | 0 | 0 | 0 | 8 | 0 | 0 | 1 | 0 |

Table C in S1 File.

| **MC** | | | | | | **MD** | | | | |
| --- | --- | --- | --- | --- | --- | --- | --- | --- | --- | --- |
| **Number of animal** | | | | | | **Number of animal** | | | | |
| **Ganglion** | **1.** | **2.** | **3.** | **4.** | **5.** | **1.** | **2.** | **3.** | **4.** | **5.** |
| **L3** | 0 | 0 | 23 | 0 | 1 | 2 | 0 | 0 | 0 | 0 |
| **L4** | 1 | 6 | 31 | 22 | 0 | 1 | 2 | 0 | 0 | 4 |
| **L5** | 9 | 21 | 30 | 32 | 169 | 1 | 7 | 0 | 3 | 23 |
| **L6** | 166 | 157 | 232 | 243 | 285 | 91 | 29 | 56 | 11 | 37 |
| **S1** | 362 | 250 | 19 | 178 | 13 | 197 | 199 | 123 | 116 | 226 |
| **S2** | 30 | 25 | 184 | 9 | 5 | 31 | 65 | 52 | 74 | 147 |
| **S3** | 21 | 2 | 8 | 0 | 1 | 10 | 15 | 6 | 0 | 20 |
| **S4** | 7 | 1 | 1 | 0 | 0 | 1 | 0 | 0 | 1 | 7 |
| **C1** | 0 | 0 | 6 | 0 | 0 | 0 | 0 | 0 | 0 | 0 |
